# Mapping prefrontal afferents along development

**DOI:** 10.3389/fnins.2026.1861524

**Published:** 2026-09-16

**Authors:** Anne Günther, Malte Stelzer, Andreas Franzelin, Annette Marquardt, Thomas G. Oertner, Ileana L. Hanganu-Opatz, Jastyn A. Pöpplau

**Affiliations:** 1 Institute of Developmental Neurosciences, Center for Molecular Neurobiology Hamburg, Hamburg Center of Neuroscience, University Medical Center Hamburg-Eppendorf, Hamburg, Germany; 2 Institute of Synaptic Physiology, Center for Molecular Neurobiology Hamburg, Hamburg Center of Neuroscience, University Medical Center Hamburg-Eppendorf, Hamburg, Germany

**Keywords:** adolescence, contralateral, corpus callosum, development, myelin, neonatal, prefrontal afferents, retrograde labeling

## Abstract

**Introduction:**

The medial prefrontal cortex (mPFC) serves as a central hub for cognitive, emotional, and social processes by integrating inputs from numerous brain regions. Understanding the mechanisms underlying the formation and myelination of prefrontal afferents is essential for elucidating the emergence, maturation, and refinement of mPFC-dependent behaviors. However, despite extensive characterization of long-range projections to the mPFC in the adult brain, their developmental trajectories remain poorly understood.

**Methods:**

In this study, we combine retrograde labeling, light-sheet imaging, and automated cell segmentation to quantitatively map afferent projections to the mPFC from neonatal stages to adulthood.

**Results:**

We show that ipsi- and contralateral afferent densities undergo marked, region-specific developmental changes. Quantitative laterality analysis revealed that many afferent pathways develop an increasing ipsilateral bias after neonatal age, arising through distinct combinations of ipsilateral and contralateral changes rather than a uniform loss of contralateral inputs. In addition, we identify region-specific developmental patterns, including increased projection densities from limbic regions during early adolescence and a progressive decline in inputs from the mediodorsal thalamus toward adulthood. Notably, myelination of interhemispheric prefrontal afferents begins only after the reduction of neonatal contralateral projections.

**Conclusion:**

Together, these findings reveal distinct, region-specific developmental dynamics of prefrontal afferents, with important implications for the maturation of mPFC-dependent functional and behavioral outputs.

## Graphical Abstract

**Figure:**
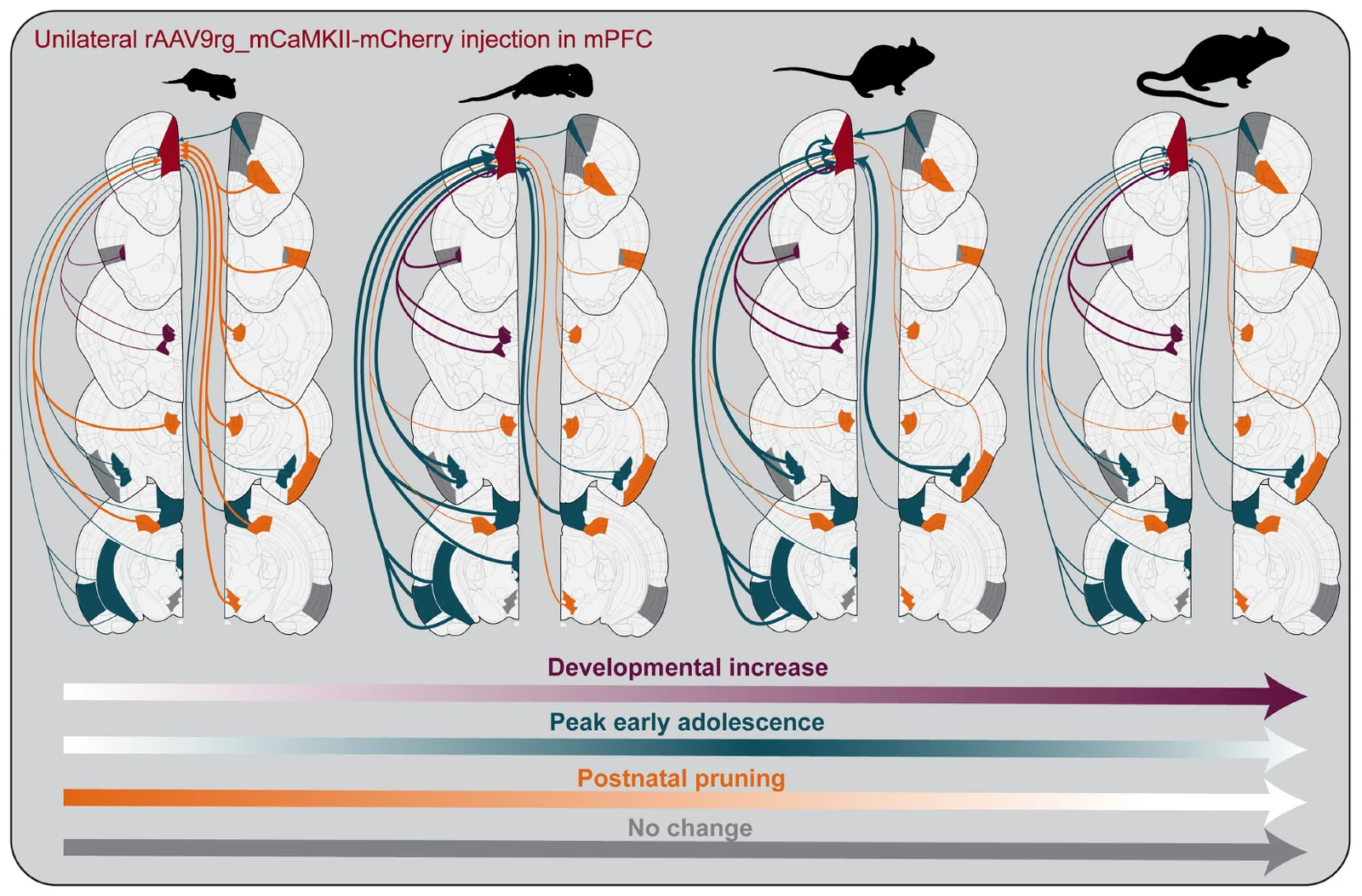
Development of ipsilateral and contralateral prefrontal afferents.

## 1 Introduction

The mPFC, a central hub of integrative processing in the brain, is involved in a wide range of cognitive, emotional, and social functions. It integrates direct axonal inputs from numerous cortical and subcortical regions through local and long-range circuits to guide behavior (Hoover and Vertes, 2007; Ährlund-Richter et al., 2019; Hanganu-Opatz et al., 2023). Within the PFC, functions are anatomically and physiologically compartmentalized, with medial subdivisions [i.e., anterior cingulate (ACA), prelimbic (PL), and infralimbic (ILA) areas] primarily contributing to sensorimotor functions, action-planning, and decision making, while ventral regions [i.e., orbitofrontal cortex (ORB)] as well as adjacent cortices [i.e., secondary motor cortices (MOs)] control emotional and motivational behaviors, respectively (Heidbreder and Groenewegen, 2003; Laubach et al., 2018; Anastasiades and Carter, 2021). In contrast, long-range inputs to the mPFC provide essential information on environmental context and internal state from other regions (Spellman et al., 2015; Burgos-Robles et al., 2017). For instance, inputs from the ventromedial thalamus (VM) to the mPFC regulate arousal states (Honjoh et al., 2018), while hippocampal projections contribute to working, recognition, and episodic memory (MacDonald et al., 2011; Takehara-Nishiuchi, 2014; Chao et al., 2017, 2020).

However, not only ipsilateral projections shape prefrontal circuits. Interhemispheric connectivity has been shown to increase the complexity of prefrontal networks (Floresco and Ghods-Sharifi, 2007; Chao et al., 2016; Wang et al., 2023). Numerous studies have investigated projections crossing the corpus callosum (CC) in rodents and humans (Yorke and Caviness, 1975; van der Knaap and van der Ham, 2011; Innocenti et al., 2022). While callosal connectivity has traditionally been assumed to be predominantly homotopic (i.e., mPFC to mPFC) (Yorke and Caviness, 1975; De Benedictis et al., 2016), more recent studies have revealed substantial heterotopy of these projections (Szczupak et al., 2023). Notably, specific functions of homotopic and heterotopic callosal connections have been described in rodents. At the homotopic level, sensory processing has been shown to be influenced by contra- and ipsilateral ACA projections (Wang et al., 2023), whereas disruptions of heterotopic pathways between the basolateral amygdala (BLA) and the ACA led to impaired decision making (Floresco and Ghods-Sharifi, 2007).

In contrast to this detailed dissection of adult prefrontal afferents, little is known about their development. Projections from other brain regions reach the mPFC during development and are thought to undergo dynamic reorganization during early life to promote maturation of higher-order cognitive (e.g., working memory) and emotional functions (e.g., fear learning) (Chini and Hanganu-Opatz, 2021). For example, projections from the mediodorsal thalamus (MD) and from the BLA progressively increase in density during development and modulate the maturation of local prefrontal circuits (Cunningham et al., 2002; Maren and Quirk, 2004; Rios and Villalobos, 2004; Parnaudeau et al., 2018; Klune et al., 2021). However, prior studies along development have focused on isolated pathways and time points, precluding a large-scale age-dependent mapping of afferents.

In this study, we addressed this knowledge gap by systematically characterizing the development of axonal projections to the mPFC from birth to adulthood. First, we combined retrograde viral tracing with light-sheet microscopy to generate a comprehensive atlas of regional afferent projection patterns to the developing mPFC. Based on these results, we used the same viral tracing strategy, combined with confocal microscopy and automated cell segmentation, to quantify projecting neurons across the brain throughout development. Additionally, we assessed the degree of myelination of retrogradely labeled inputs using immunohistochemistry. Our data provide a comprehensive developmental overview of prefrontal afferents across a broad range of brain regions, including the agranular insular cortex (AI), piriform cortex (PIRI), basolateral amygdala (BLA), basomedial amygdala (BMA), retrosplenial cortex (RSP), entorhinal cortex (ENT), ventral and intermediate CA1 (CA1v, CA1i), ventral and dorsal subiculum (SUBv, SUBd), claustrum (CLA), anterior thalamic nuclei (ATN), ventromedial thalamus (VM), mediodorsal thalamus (MD), supramammillary nucleus (SUM), periaqueductal gray (PAG), and ventral tegmental area (VTA), together with local prefrontal subdivisions. This brain-wide analysis reveals distinct region- and age-specific trajectories of mPFC afferents and provides insight into the evolving architecture of prefrontal circuits.

## 2 Materials and methods

### 2.1 Animals

All experiments were performed in compliance with German law and guidelines of the European Union for the use of animals in research (European Union Directive 2010/63/EU) and were approved by the local ethical committee (Behörde für Gesundheit und Verbraucherschutz Hamburg). Experiments were carried out in C57BL/6J mice of both sexes. Animals were housed in a 12 h light/12 h dark cycle and were given access to water and food *ad libitum*. The day of birth was considered postnatal day (P) 0.

### 2.2 Virus injections

Viral vectors were injected unilaterally into the PFC of the right hemisphere of C57BL/6J mice. Two viral vectors were applied simultaneously: rAAV9rg_mCaMKII-mCherry (#114469-AAVrg, Addgene, Watertown, MA, United States) for retrograde labeling of excitatory projection neurons and rAAV9_CAG-eGFP (#37825 Addgene, Watertown, MA, United States) for local labeling of cells at the injection site. Viral vectors were prepared in a genome copy ratio of 1:2.5 and injected at the same time using a stereotax (Kostka and Bitzenhofer, 2022; Figure 1A). Viral vectors were injected at four different time points, for which volumes and coordinates were adjusted to compensate for brain volume to result in a comparable virus spread across development, i.e., P0 (neonatal, 130 nl at a rate of 120 nl/min, 0.3 mm posterior to the inferior cerebral vein, 0.2 mm lateral to the midline and 0.4–0.8 mm ventral from the brain surface), P10 (pre-juvenile, 170 nl at a rate of 120 nl/min, 1.6 mm anterior to Bregma, 0.2 mm lateral to midline and 0.8–1.2 mm ventral from the brain surface), P20 (juvenile, 190 nl at a rate of 120 nl/min, 1.8 mm anterior to Bregma, 0.2 mm lateral to midline and 1–1.4 mm ventral from the brain surface), and P50 (adult, 210 nl at a rate of 120 nl/min, 1.9 mm anterior to Bregma, 0.2 mm lateral to midline and 1.1–1.5 mm ventral from the brain surface). Mice were transcardially perfused with 4% paraformaldehyde (PFA) 10 days after virus injection, i.e., at P10 (neonatal, *n* = 6), P20 (pre-juvenile, *n* = 6), P30 (juvenile, *n* = 6), and P60 (adult, *n* = 6). To further account for differences in viral spread at the injection site, brains were manually categorized as low spread, normal spread, or high spread. Low spread was defined as expression in only one mPFC subdivision or a very low degree of labeled cell bodies in more than one subdivision (neonatal *n* = 1, pre-juvenile *n* = 1). High spread was defined as labeled cell bodies in all three subdivisions of the mPFC, while also slightly exceeding mPFC boundaries along the dorsal-ventral or anterior-posterior axis (neonatal *n* = 1, pre-juvenile *n* = 1, juvenile *n* = 1, adult *n* = 1). Accordingly, normal spread was defined as expression in at least two subdivisions without exceeding mPFC boundaries (neonatal *n* = 4, pre-juvenile *n* = 4, juvenile *n* = 5, adult *n* = 5). Virus spread was included in the statistical analysis (see Statistical analysis, Supplementary discussion, and Statistics Supplementary Tables 1, 2).

**FIGURE 1:**
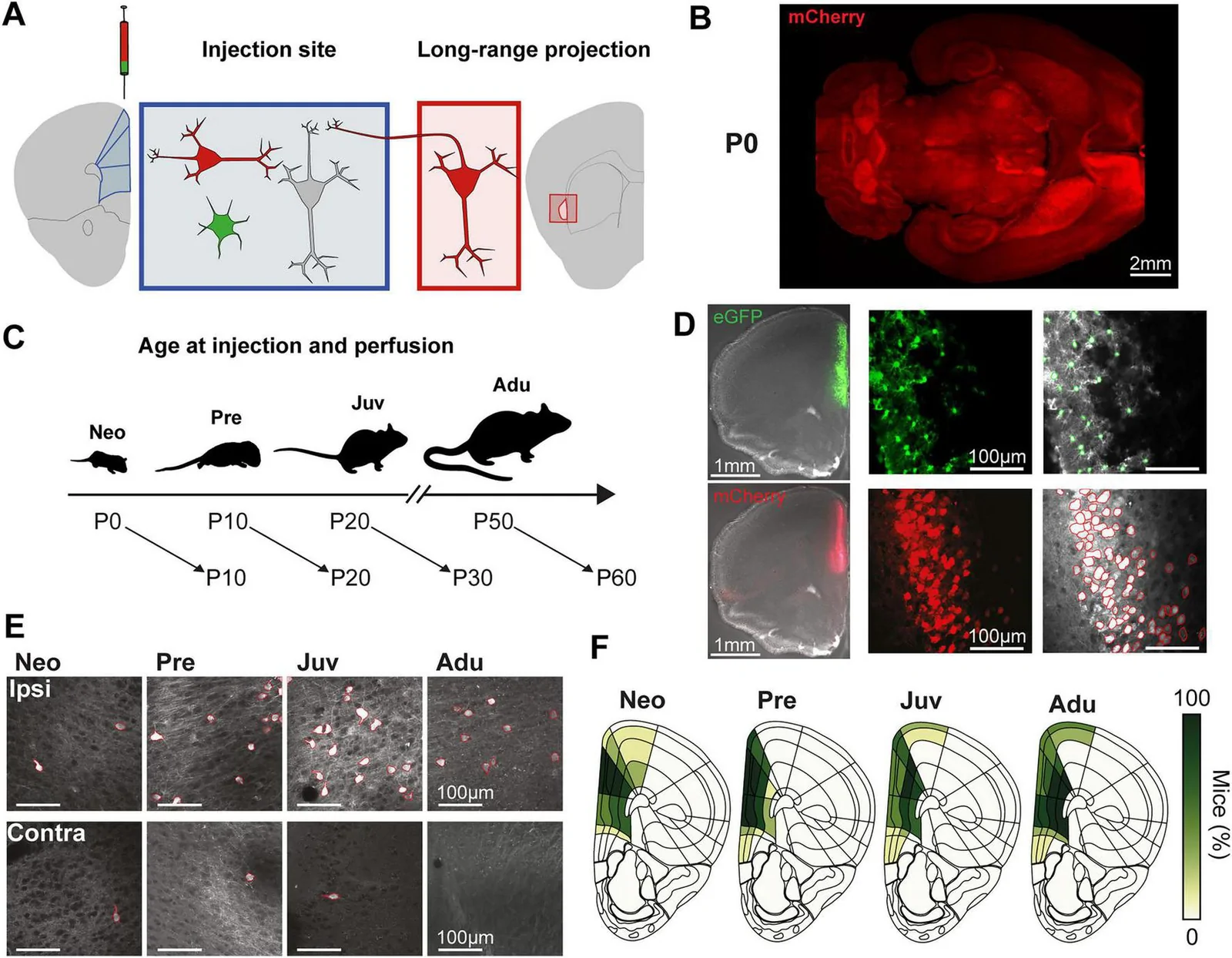
Labeling of mPFC afferents along development. **(A)** Schematic illustration of the transduction strategy with two viral vectors [rAAV9rg_mCaMKII-mCherry (red) and rAAV9_CAG-eGFP (green)] injected into the right mPFC (left), as well as an example for local and long-range targets (right). **(B)** Representative light-sheet microscopy images of a transverse section of a neonatal (injected at P0, perfused at P10) mouse with retrograde mCherry labeling (red). **(C)** Schematic of injection and perfusion time points for the four investigated age groups. **(D)** Representative fluorescence microscope images of a pre-juvenile mouse, depicting the cell detection strategy of both viral constructs at the injection site (left panel: overview, middle panel: fluorescence detected via confocal microscopy, right panel: cell segmentation). **(E)** Representative confocal images from the ipsilateral and contralateral CLA of all investigated age groups, depicting the cell detection strategy of retrogradely labeled, long-range projecting cells. **(F)** Color-coded heatmaps of viral spread across all of all investigated age groups.

### 2.3 Tissue clearing and light-sheet imaging

Light-sheet imaging on an open-source mesoSPIM (Voigt et al., 2019) was used to generate brain-wide overviews of mPFC-projecting brain regions at neonatal (injected at P0, perfused at P10) and pre-juvenile (injected at P10, perfused at P20) age. Brains were fixed by perfusion before being cleared with SHIELD reagent (Park et al., 2019) to preserve endogenous fluorescence and imaged in EasyIndex solution (RI = 1.465, Life Canvas Technologies) with an Orca Flash 4.0 camera (Hamamatsu).

### 2.4 Cell quantification

For confocal microscopy, brains were prepared in coronal 100 μm sections and mounted on slides using Vectashield with DAPI (Vector Laboratories, Burlingame, CA, United States). For cell quantification, images were obtained using a confocal microscope (LSN700, Zeiss, Germany) with a 20× objective (2,048 × 2,048 pixels; 319.5 × 319.5 μm). Areas were demarcated according to the coronal P56 mouse reference atlas from the Allen Brain Atlas^1^. Additionally, images of mice injected at P0 were also compared to a developing mouse brain atlas (Paxinos, 2007). Depending on the size of the targeted brain region, between 3 and 6 images were acquired per mouse. Images were segmented with Cellpose 2 in Anaconda (Python 3.8) using both the standard model algorithm “cyto2” for the images of cells transduced by rAAV9rg_CaMKII-mCherry and a self-trained algorithm as described for the human-in-the-loop approach (Pachitariu and Stringer, 2022) for rAAV9_CAG-eGFP-transduced cells. After automatic cell segmentation, all images were validated by visual inspection. Confocal images from 22 prefrontal and long-range projecting brain sections with mCherry-labeled neurons were used to quantify the density of retrogradely labeled mPFC-projecting neurons across development. Images of eGFP-labeled cells were acquired from prefrontal areas at the injection site (ACA, PL, ILA, ORB, MOs) for normalization of quantification of retrogradely labeled projections. Normalized cell count (NCC) was calculated per brain area using mCherry-labeled cells of a single image from each brain area of an animal (r) and all eGFP-labeled cells of an animal (ḡ) as:


$$N\,C\,C=\frac{r}{r+\bar{g}}$$

To quantify hemispheric bias in afferent labeling, an ipsilateral-contralateral laterality index was calculated for each animal as:


$$\text{Laterality}\,\text{Index}=\frac{{\text{N}}_{\text{ipsi}}-{\text{N}}_{\text{contra}}}{{\text{N}}_{\text{ipsi}}+{\text{N}}_{\text{contra}}}$$

where N_*ipsi*_ and N_*contra*_ represent the number of labeled cells in the hemisphere ipsilateral and contralateral to the injection site, respectively. Positive values indicate a predominance of ipsilateral labeling, negative values indicate greater contralateral labeling, and values around zero reflect approximately balanced bilateral afferent labeling. As an additional parameter, the mean cell count (x) was calculated as the mean of mCherry-labeled cells from all sections of one region for all animals from all age groups.

### 2.5 Immunohistochemistry

For immunohistochemistry, brains were sectioned coronally at 50 μm thickness. Staining of myelin basic protein (MBP) at the CC was performed on the corresponding sections of injected brains after perfusion at P10 (*n* = 4), P20 (*n* = 3), P30 (*n* = 3), and P60 (*n* = 3) as previously described (Günther et al., 2026; Parbst et al., 2025). Briefly, brain slices were permeabilized using 0.8% Triton X-100 (Sigma-Aldrich, MO, USA) and blocked with 5% normal goat serum and 5% normal donkey serum (Jackson Immuno Research, PA, USA) for 1 h. Samples were incubated overnight at 4 °C with an MBP antibody (1:500, Abcam ab40390; Cat# ab40390, RRID:AB_114152). As a secondary antibody Goat-Anti-Rabbit IgG (H&L) DyLight 633 (1:1000, Novus, NBP1-76057; Cat# NBP1-76057, RRID:AB_11059121) was used.

### 2.6 Myelin quantification

For myelin quantification, images were obtained using a confocal microscope (F1000, Olympus, Tokyo, Japan) with a 60× objective and 2× zoom (1024 × 1024 pixels; 105.47 × 105.47 mm). Immunohistochemically stained samples from injected brains were used to image axonal fibers and myelin at the CC. Confocal image stacks were consistently acquired at the midline of the corpus callosum from anatomically comparable rostrocaudal sections across animals (i.e., P10 +0.6 AP, P20 +0.7 AP, P30 +0.7 AP, and P60 +1.0 AP). For every animal, 3–4 stacks from different brain slices (10–30 single images, 150 nm interval) were acquired. After preprocessing and thresholding in ImageJ, colocalization of MBP-positive and mCherry-labeled structures was calculated in Matlab R2023a using a custom-written algorithm similar to Pöpplau et al. (2024). Values were normalized for stack depth.

### 2.7 Statistical analysis

Statistical analysis was carried out in R using linear mixed effect models (LME) of the “lme4” package in R 4.4.1. We assessed the impact of age on NCCs while including sex and viral spread as additional fixed effects. Animal identity was included as a random intercept to account for multiple image-based measurements obtained from the same individual animal. Analyzed image stacks of myelinated structures, labeled axonal fibers, and their colocalization were similarly analyzed with LME models including age, sex, viral spread, and the number of images within each analyzed stack as fixed effects, and animal identity as a random effect (full model: NCC ∼ age + viral spread + sex + (1 | animal)). The number of images within each stack was included as an additional covariate to account for potential variation in the amount of tissue sampled between image stacks and significantly influenced the measured values (see Statistics Supplementary Table 1). Briefly, likelihood ratio tests were performed to compare log-likelihoods between the full and the reduced model. To test for a significant effect of age group, sex, viral spread, or, for the myelination analysis, the number of stack images, each fixed effect was individually removed from the full model, and the resulting reduced model was compared with the full model using the “anova” function. Post-hoc pairwise comparisons between age groups were conducted using Tukey-adjusted pairwise contrasts implemented with the “multcomp” package. *P*-values were adjusted using the Holm method “holm” to account for multiple comparisons (see Statistics Supplementary Table 1).

## 3 Results

### 3.1 mPFC afferents show age-dependent trajectories

Resolving the developmental dynamics of long-range projections to the mPFC from different brain regions and between hemispheres is the basis for understanding how prefrontal circuits gain and develop their functions. In a first step, we used light-sheet microscopy on cleared brain tissue to obtain a whole-brain overview of mPFC afferents during early postnatal development. We focused this exploratory analysis on the earliest developmental stages because the organization of adult mPFC afferents has already been comprehensively characterized (Ährlund-Richter et al., 2019). For the qualitative light-sheet overview, one mouse received unilateral mPFC injections of rAAV9rg_mCaMKII-mCherry and rAAV9_CAG-eGFP at P0, and a second mouse received the same injections at P10, following a previously established protocol for assessing afferent development in the olfactory bulb (Kostka and Bitzenhofer, 2022). Tissue was perfused and cleared 10 days after injection (P10 and P20, respectively; Figures 1A, B and Supplementary Figure 1). This qualitative whole-brain screening was used to identify candidate afferent regions for subsequent quantitative confocal analysis across the full developmental range. Based on this, we selected 22 brain regions with direct axonal projections to the mPFC during early development and adulthood for detailed analysis. We also included the PAG and VTA, which showed prominent glutamatergic afferent projections during development, which is not reported for the adult brain (Hoover and Vertes, 2007; Ährlund-Richter et al., 2019). For quantification, we used confocal microscopy on slices from injected brains across the entire developmental period (i.e., neonatal P0, pre-juvenile P10, juvenile P20, adult P50). We then performed cell segmentation (Figures 1C–E) to quantify the density of retrogradely labeled mPFC-projecting neurons in prefrontal subdivisions (ACA, PL, ILA, ORB, MOs) as well as in cortical (AI, PIRI, BLA, BMA, RSP, ENT, CA1v, CA1i, SUBv, SUBd) and subcortical areas (CLA, ATN, VM, MD, SUM, PAG, VTA), projecting ipsi- and contralaterally to the mPFC. We calculated the NCC (normalized cell count: calculated as the ratio of mCherry-labeled cells from each brain area vs. all eGFP- and mCherry-labeled cells, see section “2 Materials and methods”) to normalize for differences in local viral spread across age groups (Figure 1F, see Statistics Supplementary Tables 1, 2, and Supplementary discussion).

### 3.2 mPFC afferent organization increases along development

In order to assess local prefrontal afferent labeling, we quantified retrogradely labeled cells in prefrontal subdivisions of the ipsilateral hemisphere (marked as *^i^* for all regions). The density of labeled cells in mPFC and adjacent subdivisions changed with age. ORB*^i^* labeling was influenced by sex and viral spread (see Figures 2A–E and Statistics Supplementary Table 1, 2). For the ACA*^i^* and PL*^i^* the NCC increased from neonatal to pre-juvenile age and remained stable afterward (Figures 2A,B). Solely ILA*^i^* local afferent labeling peaked at juvenile age (Figure 2C). In contrast, the NCC in adjacent MOs*^i^* and ORB*^i^* did not significantly change with age (Figures 2D,E). Taken together, these data indicate that retrogradely labeled inputs originating from ACA*^i^*, PL*^i^*, and ILA*^i^* become more prominent during development.

**FIGURE 2:**
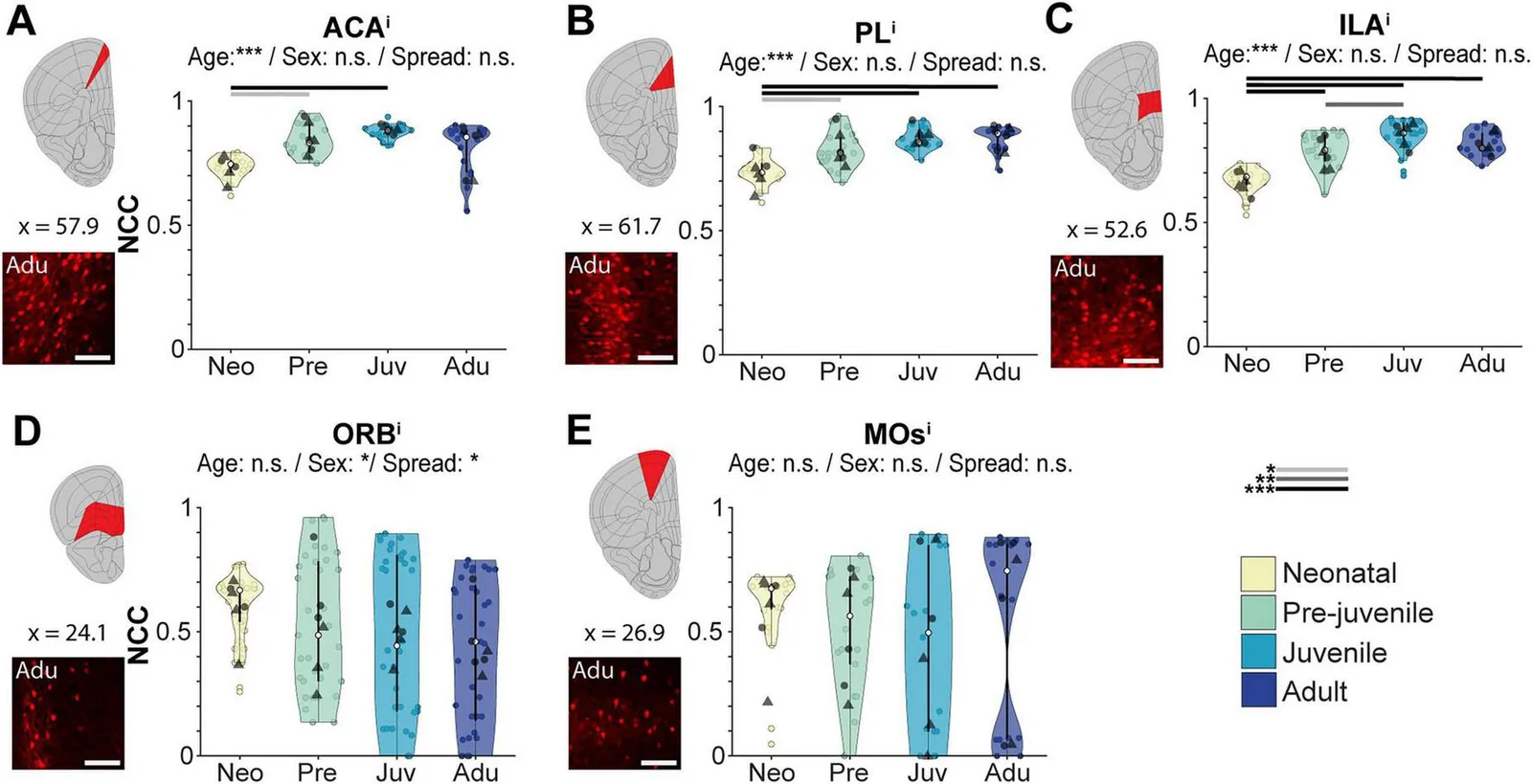
Age-dependent dynamics of local prefrontal afferent labeling. **(A)** Violin plots displaying the NCC of prefrontally projecting ACA^i^ cells (*n* = 72 images, 24 mice) for each age group. **(B)** Same as **(A)** for PL^i^ (*n* = 67 images, 24 mice). **(C)** Same as **(A)** for ILA^i^ (*n* = 83 images, 24 mice). **(D)** Same as **(A)** for ORB^i^ (*n* = 136 images, 24 mice). **(E)** Same as **(A)** for MOs^i^ (*n* = 70 images, 24 mice). Ipsilateral areas are indicated in red in schematics on the left with mean cell count given as x below sections. Small colored dots indicate individual image-level measurements, whereas larger black circles and triangles indicate the mean NCC of individual male and female mice, respectively. For each region, representative example images of adult mice are shown as a quantitative reference. Violin plots are represented as median with 25th to 75th percentile. See Statistics Supplementary Table 1 for details. Significant differences are indicated as *, **, *** for *p* < 0.05, 0.01, 0.001, respectively.

### 3.3 Cortical long-range afferents to the mPFC peak at (pre-)juvenile age

Next, we quantified the density of long-range afferents originating from ipsilateral cortical regions (RSP*^i^*, PIRI*^i^*, AI*^i^*, ENT*^i^*, SUBv*^i^*, SUBd*^i^*, CA1i*^i^*, and CA1v*^i^*) as well as adjacent regions (BLA*^i^*, BMA*^i^*, and CLA*^i^*). While viral spread had no significant effect on labeling, sex significantly influenced labeling in the CLA*^i^*, RSP*^i^*, and PIRI*^i^*, with female mice showing lower labeling across age groups (see Statistics Supplementary Tables 1, 2). Except for AI*^i^*, which exhibited consistently high afferent density across development (Figure 3A), most regions showed distinct age-dependent afferent labeling patterns. Projections from BMA*^i^* and CLA*^i^* increased markedly from neonatal to pre-juvenile age and changed only modestly thereafter. A similar, although less pronounced, developmental pattern was observed for ENT*^i^* and SUBv*^i^*. Notably, BLA*^i^*-to-mPFC afferents were nearly absent at neonatal age, emerged by pre-juvenile age, and reached their highest density at juvenile age. In contrast, RSP*^i^* afferent density peaked at pre-juvenile age, whereas CA1i*^i^* and CA1v*^i^* projections peaked at juvenile age and remained comparatively low at the other developmental stages (Figures 3B–I,K). SUBd*^i^* displayed a distinct developmental trajectory, with dense projections to the mPFC at neonatal age that progressively declined and were nearly absent in adulthood (Figure 3J).

**FIGURE 3:**
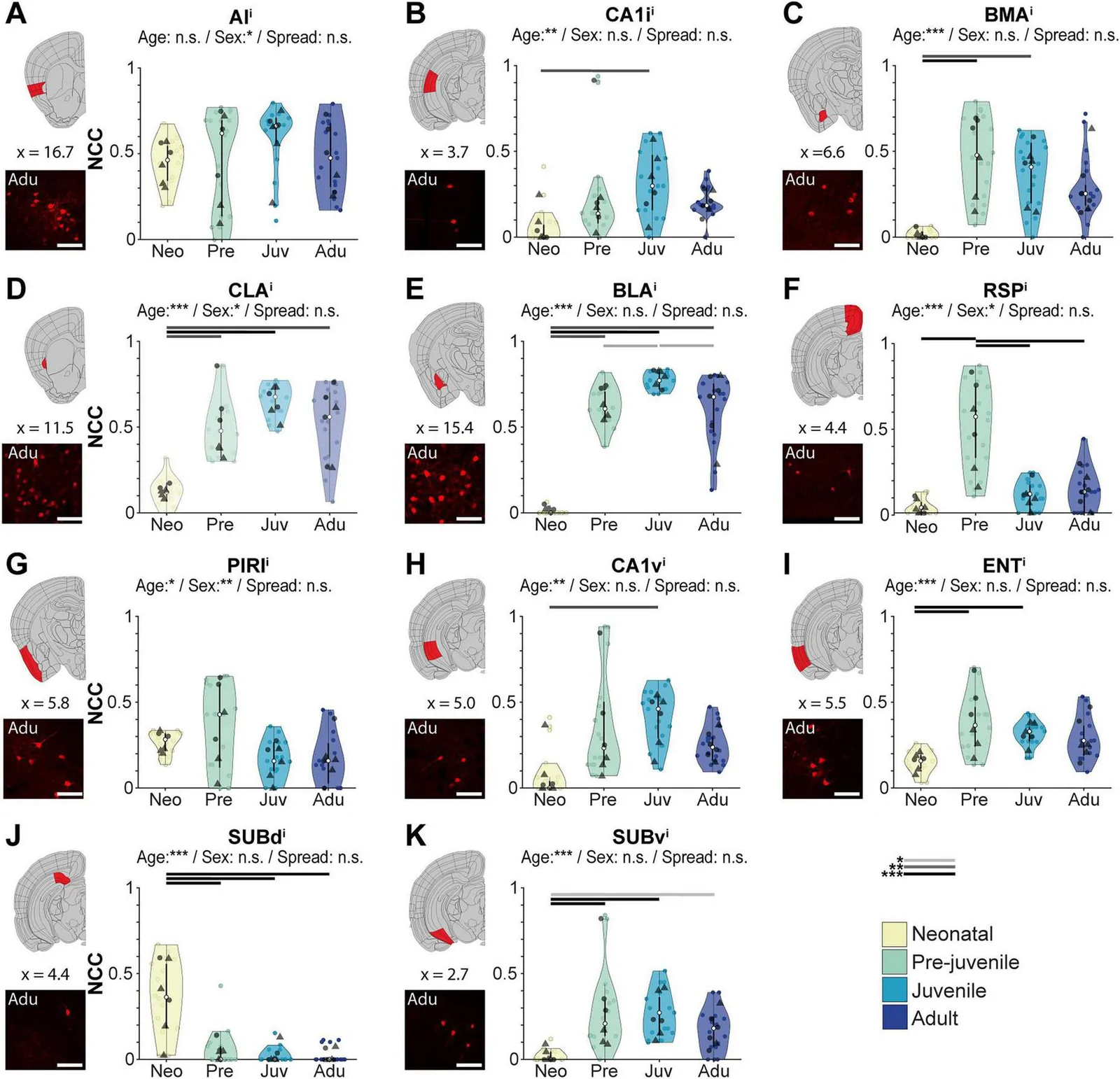
Age-dependent dynamics of cortical and subcortical projections targeting the ipsilateral mPFC. **(A)** Violin plots displaying the NCC of prefrontally projecting AI^i^ cells (*n* = 68 images, 24 mice) for each age group. **(B)** Same as **(A)** for CA1i^i^ (*n* = 67 images, 24 mice). **(C)** Same as **(A)** for BMA^i^ (*n* = 72 images, 24 mice). **(D)** Same as **(A)** for CLA^i^ (*n* = 67 images, 24 mice). **(E)** Same as **(A)** for BLA^i^ (*n* = 70 images, 24 mice). **(E)** Same as **(A)** for BLA^i^ (*n* = 61 images, 24 mice). **(F)** Same as **(A)** for RSP^i^ (*n* = 70 images, 24 mice). **(G)** Same as **(A)** for PIRI^i^ (*n* = 64 images, 24 mice). **(H)** Same as **(A)** for CA1v^i^ (*n* = 69 images, 24 mice). **(I)** Same as **(A)** for ENT^i^ (*n* = 64 images, 24 mice). **(J)** Same as **(A)** for SUBd^i^ (*n* = 70 images, 24 mice). **(K)** Same as **(A)** for SUBv^i^ (*n* = 64 images, 24 mice). Assessed ipsilateral areas are indicated in red in schematics on the left, with mean cell count given as x below sections. Small colored dots indicate individual image-level measurements, whereas larger black circles and triangles indicate the mean NCC of individual male and female mice, respectively. For each region, representative example images of adult mice are shown as a quantitative reference. Violin plots are represented as median with 25th to 75th percentile. See Statistics Supplementary Table 1 for details. Significant differences are indicated as *, **, *** for *p* < 0.05, 0.01, 0.001, respectively.

Together, these findings reveal distinct region-specific developmental dynamics of ipsilateral inputs to the mPFC, with labeling of long-range mPFC-projecting populations being particularly dense during the pre-juvenile and juvenile periods.

### 3.4 Prefrontal projections from thalamic and hypothalamic nuclei develop in an inverse manner

Next, we analyzed long-range prefrontal afferents arising from ipsilateral subcortical brain regions (ATN*^i^*, VM*^i^*, MD*^i^*, SUM*^i^*, PAG*^i^*, and VTA*^i^*). For the ATN*^i^*, we quantified the interanterodorsal nucleus as well as ventral and dorsal sections of the AM. VTA*^i^*-to-mPFC projections remained relatively stable throughout development (Figure 4A). In contrast, afferent densities from VM*^i^* and ATN*^i^* increased from neonatal to pre-juvenile age and remained largely stable thereafter, although neonatal projection densities were low in VMi but already high in ATN*^i^* (Figures 4B,C). MD*^i^* afferent density strongly decreased with age, indicating a progressive sparsification of prefrontal inputs from this region (Figure 4D). SUM*^i^*-to-mPFC projections showed a similar decline, although this decrease emerged only from pre-juvenile age onward (Figure 4E). Notably, projections from both VM*^i^* and SUM*^i^* were influenced by sex. Consistent with the pattern observed in several cortical regions, SUM*^i^* afferent densities were lower in females across age groups. In contrast, females showed higher VM*^i^* afferent densities than males after neonatal age (see Statistics Supplementary Tables 1, 2 and Supplementary discussion). PAG*^i^*-to-mPFC projections were generally sparse but followed a developmental pattern similar to that observed in several cortical regions, with afferent density peaking at pre-juvenile age (Figure 4F).

**FIGURE 4:**
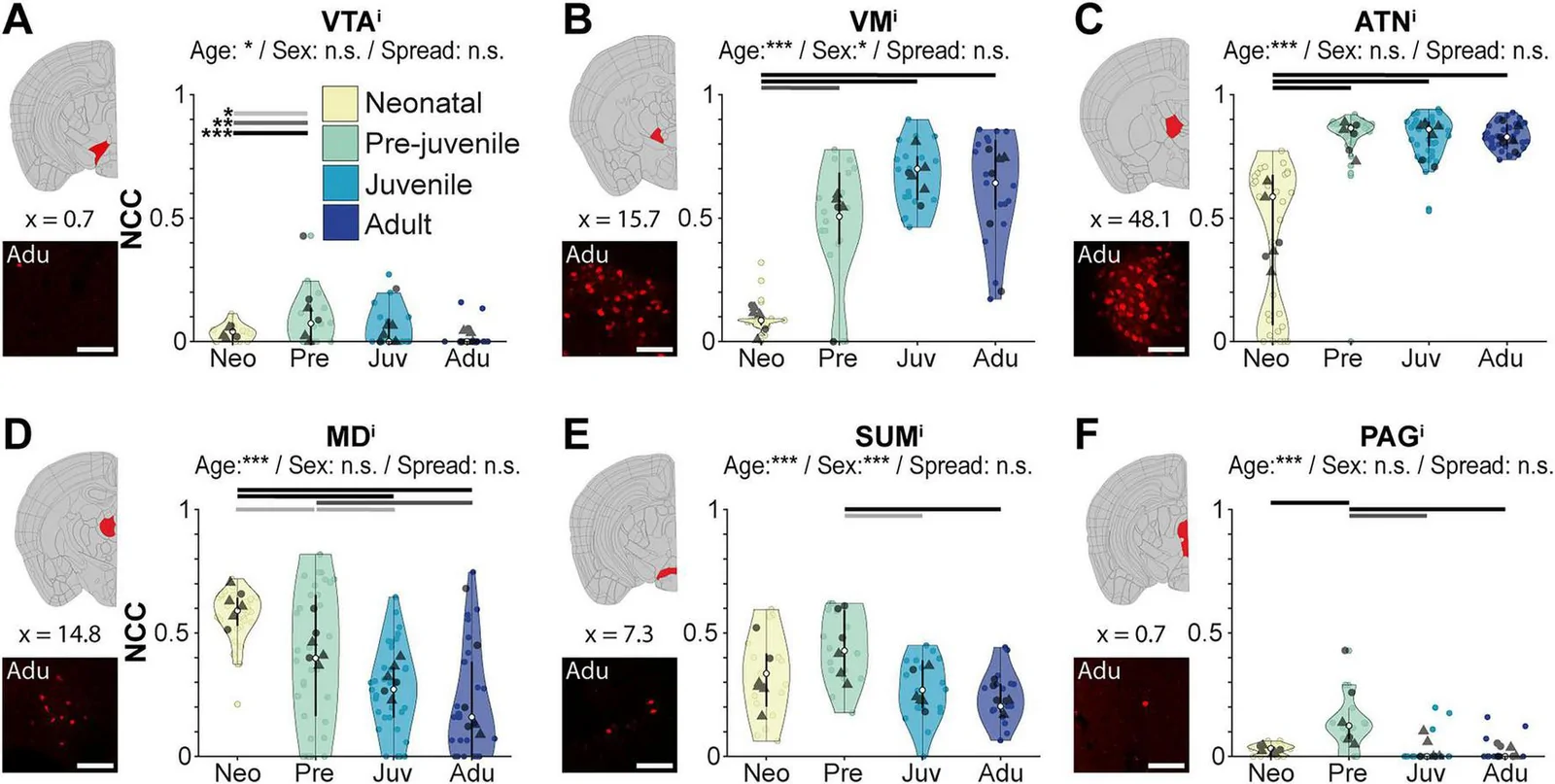
Age-dependent dynamics of projections from midbrain areas targeting the ipsilateral mPFC. **(A)** Violin plots displaying the NCC of prefrontally projecting VTA^i^ cells (*n* = 63 images, 23 mice) for each age group. **(B)** Same as **(A)** for VM^i^ (*n* = 71 images, 24 mice). **(C)** Same as **(A)** for ATN^i^ (*n* = 137 images, 24 mice). **(D)** Same as **(A)** for MD^i^ (*n* = 135 images, 24 mice). **(E)** Same as **(A)** for SUM^i^ (*n* = 68 images, 24 mice). **(F)** Same as **(A)** for PAG^i^ (*n* = 62 images, 24 mice). Assessed ipsilateral areas are indicated in red in schematics on the left with mean cell count given as x below sections. Small colored dots indicate individual image-level measurements, whereas larger black circles and triangles indicate the mean NCC of individual male and female mice, respectively. For each region, representative example images of adult mice are shown as a quantitative reference. Violin plots are represented as median with 25th to 75th percentile. See Statistics Supplementary Tables 1, 2 for details. Significant differences are indicated as *, **, *** for *p* < 0.05, 0.01, 0.001, respectively.

In summary, most prefrontal afferents from subcortical regions reach high densities at neonatal or pre-juvenile age, yet their dynamics, either increasing or decreasing with age, are region-specific.

### 3.5 Increasing ipsilateral dominance of prefrontal afferents

Adult mPFC has been reported to receive afferents from numerous contralateral regions (Anastasiades and Carter, 2021). To clarify how these projections evolve along development, we monitored contralateral prefrontal afferents from birth to adulthood alongside ipsilateral projections (marked as *^c^* for all regions). No age-dependent changes of prefrontal afferent density from contralateral ACA*^c^*, PLc, ILAc and MOs*^c^* were detected (Supplementary Figures 2A,B). In contrast, the number of labeled cells in the ORB*^c^* decreased from neonatal to juvenile and adult age, following a similar trend as observed for the ORB*^i^* (Supplementary Figure 2B). Based on their developmental trajectories, we classified contralateral long-range afferents into four distinct patterns. First, afferents were prominent early in development and strongly declined thereafter. This pattern was observed for projections from AI*^c^*, ATN*^c^*, MD*^c^*, SUM*^c^*, SUBd*^c^*, PIRI*^c^*, VTA*^c^*, and RSP*^c^* (Figures 5A,C,E and Supplementary Figures 2C,D). Among these regions, VTA*^c^* and RSP*^c^* were unique in that retrogradely labeled cells were present at neonatal and pre-juvenile age before subsequently declining (Supplementary Figure 2D). Second, some contralateral afferents were already present at neonatal age and remained relatively stable throughout development, as observed for projections from CLA*^c^* and ENT*^c^* (Figures 5B,D and Supplementary Figure 2E). Third, BLA*^c^* and BMA*^c^* projections displayed a distinct developmental profile, with afferent densities peaking at juvenile age (Supplementary Figure 2F). Fourth, several regions showed little to no contralateral prefrontal labeling throughout development, including VM*^c^*, PAG*^c^*, CA1v*^c^*, CA1i*^c^*, and SUB*^c^* (Figure 5D and Supplementary Figures 2G,H). Notably, labeling in several contralateral regions was significantly influenced by viral spread (see Statistics Supplementary Tables 1, 2 and Supplementary discussion), whereas among ipsilateral regions such an effect was detected only in the ORB. This difference may arise because contralateral projections are generally sparser and more topographically restricted, making their quantification more sensitive to variation in the viral spread at the injection site.

**FIGURE 5:**
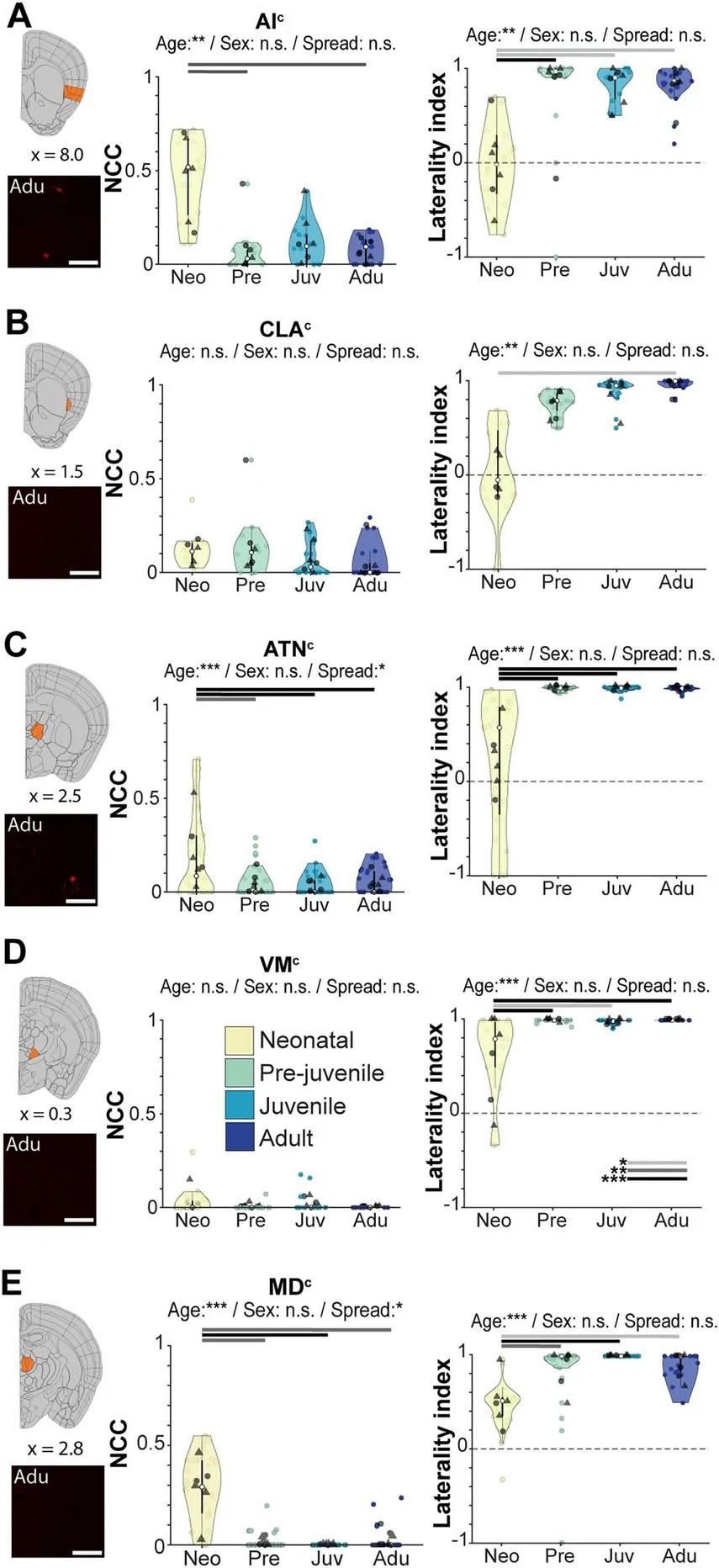
Age-dependent dynamics of contralateral mPFC afferent labeling. **(A)** Left, violin plots displaying the NCC of prefrontally projecting AI^c^ cells. Right, Laterality index of ipsi-contralateral densities (*n* = 64 images, 24 mice) for each age group. **(B)** Same as **(A)** for CLA^c^ (*n* = 69 images, 23 mice). **(C)** Same as **(A)** for ATN^c^ (*n* = 135 images, 24 mice). **(D)** Same as **(A)** for VM^c^ (*n* = 69 images, 24 mice). **(E)** Same as **(A)** for MD^c^ (*n* = 132 images, 24 mice). Assessed contralateral areas are indicated in orange in schematics on the left with mean cell count given as x below sections. Small colored dots indicate individual image-level measurements, whereas larger black circles and triangles indicate the mean NCC of individual male and female mice, respectively. For each region, representative example images of adult mice are shown as a quantitative reference. Violin plots are represented as median with 25th to 75th percentile. See Statistics Supplementary Tables 1, 2 for details. Significant differences are indicated as *, **, *** for *p* < 0.05, 0.01, 0.001, respectively.

To further assess the hemispheric bias of mPFC afferent organization across development, we calculated an ipsilateral-contralateral laterality index, with positive values indicating a predominance of ipsilateral labeling. Despite distinct developmental trajectories of ipsilateral and contralateral afferents, the laterality index generally increased after neonatal age, indicating a developmental shift toward greater ipsilateral bias. For example, AI*^i^* and ATN*^i^* afferent densities remained relatively stable across development, whereas their contralateral counterparts declined after neonatal age, resulting, as expected, in an increased ipsilateral bias (Figures 5A,C). Conversely, CLA*^c^* and VM*^c^* showed little developmental change, whereas their ipsilateral counterparts increased with age, resulting in a progressively stronger ipsilateral bias across development (Figures 5B,D). Finally, the MD showed age-dependent decreases in labeling in both hemispheres. However, the decline was more pronounced contralaterally, similarly resulting in an increasing ipsilateral bias across development (Figure 5E).

Together, these findings reveal pronounced region-specific developmental dynamics of contralateral prefrontal afferents and demonstrate a general shift toward greater ipsilateral predominance of afferent labeling after neonatal age. Notably, the balance between ipsilateral and contralateral inputs develops in a region-specific manner, resulting in distinct patterns even between closely related structures such as different thalamic nuclei.

### 3.6 Myelination of contralateral prefrontal afferents emerges after neonatal age

In addition to monitoring the density of contralateral prefrontal afferents, we also assessed their myelination. For this, we focused on the cortico-cortical projections crossing the CC. The CC represents the largest white matter tract in the mammalian brain (Fitsiori et al., 2011; De León Reyes et al., 2020; Szczupak et al., 2021), and it has been extensively studied at adult age (Yorke and Caviness, 1975; van der Knaap and van der Ham, 2011; De León Reyes et al., 2020; Innocenti et al., 2022). We characterized the developmental dynamics of myelination of contralateral prefrontal afferents in the previously defined age groups (i.e., neonatal, pre-juvenile, juvenile, and adult) (Figure 6A). Overall, myelin expression increased with age (Figure 6B). Retrogradely labeled axons that colocalized with myelin were sparse in neonatal mice, but their density strongly increased at pre-juvenile age and remained elevated until adulthood (Figure 6C). Similarly, the portion of myelin colocalizing with labeled axons increased from neonatal to adult age (Figure 6D).

**FIGURE 6:**
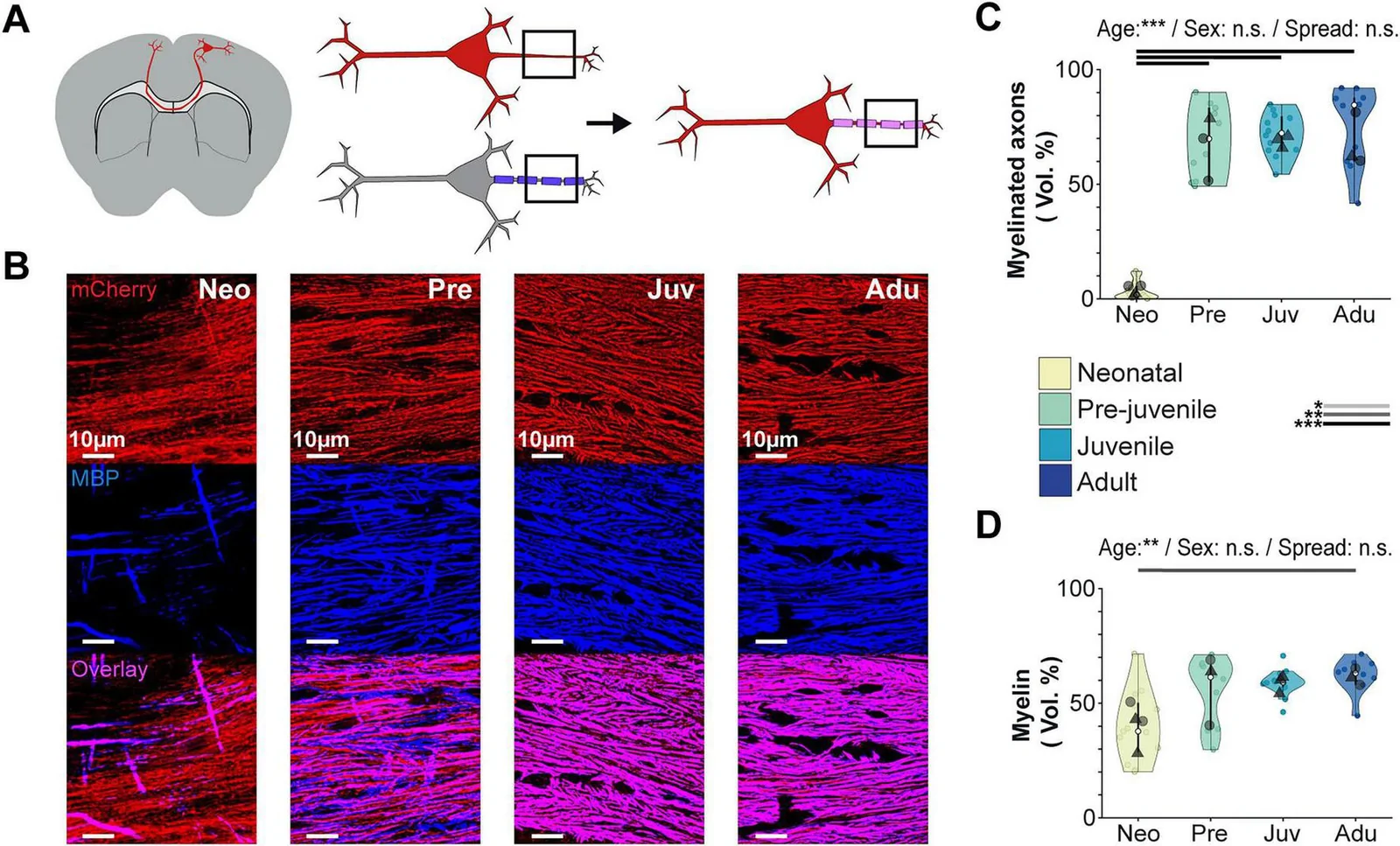
Myelination of hemisphere-crossing prefrontal afferents across age groups. **(A)** Schematic illustration of myelination quantification strategy of prefrontal afferents crossing the CC. Confocal image stacks (black frames) were acquired from CC, showing mCherry-labeled prefrontal afferents (red) and immunohistochemically stained myelin sheaths (MBP, blue). Image stacks were overlaid to analyze colocalization (magenta). **(B)** Representative confocal images of labeled axons and myelin sheaths in the CC. Labeled axons (red) and immunohistochemically stained MBP (blue), as well as their overlay (magenta), are shown for each age group. **(C)** Violin plots displaying the volumetric percentage of MBP-overlaid prefrontal afferents normalized to the total volume of mCherry-labeled axons for each age group. **(D)** Violin plots displaying the percentage of MBP colocalizing with mCherry-labeled axons normalized to the total volume of MBP for each age group (*n* = 45 images, 13 mice). Violin plots are represented as median with 25th to 75th percentile. Small colored dots indicate individual image-level measurements, whereas larger black circles and triangles indicate the mean NCC of individual male and female mice, respectively. See Statistics Supplementary Table 1 for details. Significant differences are indicated as *, **, *** for *p* < 0.05, 0.01, 0.001, respectively.

## 4 Discussion

The PFC is a higher-order cortical region that is essential for complex cognitive and affective processes such as decision making, working memory, and emotional regulation. Its maturation is protracted when compared to primary sensory cortices (Chini and Hanganu-Opatz, 2021), suggesting a prolonged time window for structural and functional refinement of prefrontal circuits. In this study, we unraveled the developmental dynamics of local and long-range ipsilateral and contralateral projections to the mPFC, with a specific focus on developmental changes in the density and hemispheric distribution of retrogradely labeled mPFC-projecting neurons from different brain regions (i.e., ACA, PL, IL, ORB, MOs, AI, PIRI, BLA, BMA, RSP, ENT, CA1v, CA1i, SUBv, SUBd, CLA, ATN, VM, MD, SUM, PAG, and VTA) as well as the onset of myelination. Our findings reveal regionally distinct maturational dynamics of prefrontal afferents that might underlie functional and behavioral development.

### 4.1 Developmental shift toward ipsilateral dominance of projections to the mPFC

At neonatal age, afferent projections to the mPFC show a comparatively strong bilateral organization. However, hemispheric refinement of prefrontal inputs emerges through distinct region-specific developmental trajectories rather than through a uniform loss of contralateral afferents. The most commonly observed dynamic of contralateral projections was a decline in projection density after neonatal age (i.e., ORB^c^, AI^c^, PIRI^c^, SUBd^c^, ATN^c^, MD^c^, SUM^c^, VTA^c^, and RSP^c^). While postnatal pruning of perinatal contralateral projections for the ATN and MD has been reported (Minciacchi and Granato, 1989; De León Reyes et al., 2019), our laterality analysis extends these observations by demonstrating that the balance of prefrontal afferents shifts toward the ipsilateral hemisphere after neonatal age across cortical and subcortical pathways. This suggests that the refinement of interhemispheric afferent labeling represents a widespread, yet regionally heterogeneous, feature of mPFC maturation.

Our data further show that myelination of CC-crossing prefrontal afferents is sparse at neonatal age and strongly increases from pre-juvenile age onward, coinciding with the emergence of contralateral afferent refinement. In adult animals, the CC, is the largest white matter tract in the mammalian brain (Fitsiori et al., 2011; De León Reyes et al., 2020) and contains mostly cortico-cortical fibers (Szczupak et al., 2021), although evidence also suggests the presence of cortico-subcortical connections (Milardi et al., 2015; De Benedictis et al., 2016). In line with previous studies (Sturrock, 1980), we detected low amounts of myelin in the CC and found that mPFC axonal fibers are largely unmyelinated at neonatal age. As has been previously shown (Paus et al., 1999; Fornari et al., 2007), myelination of prefrontal afferents increases strikingly during the second postnatal week, co-occurring with weaning and the onset of more complex behavioral abilities (Klune et al., 2021). As oligodendrocyte engagement and axonal wrapping are promoted by activity-dependent mechanisms (de Faria et al., 2019), the absence of myelin in early-stage interhemispheric fibers supports the idea that these projections might lack the necessary activity for functional embedding into mature circuits. In line with this hypothesis, the emergence of activity follows a protracted development in the mPFC (Brockmann et al., 2011). This transient bilateral connectivity may serve as a scaffolding mechanism for the maturation of local prefrontal microcircuits, facilitating early synchronization across hemispheres. The question of whether early contralateral prefrontal projections are functionally active remains to be addressed. Alternatively, these projections could reflect developmental remnants of cerebral ontogenesis that, in the absence of a specific function, are pruned with ongoing maturation.

Developmental refinement was not restricted to contralateral afferents. Ipsilateral inputs followed similarly heterogeneous trajectories: local ACA and PL afferent labeling as well as inputs from VM and ATN increased from neonatal to pre-juvenile age and subsequently stabilized, whereas other projections were already established early or underwent a progressive reduction. Most strikingly, MD^i^ and SUBd^i^ afferents decreased substantially with age. This result partially contradicts earlier qualitative, not statistically validated reports that suggested a rebound of MD input in adulthood (Rios and Villalobos, 2004). Given the well-established role of the MD in working memory and cognitive functions (Parnaudeau et al., 2018), developmental pruning of its projections to the mPFC appears paradoxical. However, pruning of excessive, non-informative, or redundant projections might improve signal fidelity. Our data suggest that this refinement of projections to the mPFC continues into adulthood, potentially facilitating the emergence of precise, goal-directed behavior.

Notably, these developmental trajectories should not necessarily be interpreted as reflecting the initial arrival of afferent axons in the mPFC. Several major afferent systems are already established during early postnatal life and undergo substantial subsequent refinement. For example, MD afferents are present from birth and are reorganized during the first postnatal weeks (Rios and Villalobos, 2004), whereas amygdala-prefrontal projections continue to expand during juvenile and adolescent development (Cunningham et al., 2002). Hippocampal-prefrontal projections are likewise present early in life and undergo prolonged structural and functional maturation (Song et al., 2022). Thus, age-dependent changes in retrograde labeling likely reflect growth, stabilization, or pruning of established pathways rather than simply their initial emergence.

### 4.2 Afferent labeling to the mPFC peaks during early adolescence

One of the most salient observations in our data is the pronounced maturation of multiple long-range projections to the mPFC during the pre-juvenile and juvenile periods, coinciding with the emergence of higher-order cognitive functions (Klune et al., 2021; Thies et al., 2025). The developmental increase was not uniform across mPFC-projecting regions. Some inputs, such as those from BMA, CLA, ENT, SUBv, VM, and ATN, increased strongly by pre-juvenile age. RSP^i^ projections peaked at pre-juvenile age, while afferents from CA1^i^, CA1v^i^, and BLA^i^, as well as contralateral BLA and BMA projections, reached their highest densities at juvenile age. This maturation of afferents during adolescence is notable given their roles in memory, rewards, and adaptive behavior. For example, the SUBv is known to be involved in the rewards circuit of the brain (Ding et al., 2020) and a role of the CA1 in anticipation of consequences has been proposed (Fyhn et al., 2002; Numan, 2015). Thus, this temporal alignment of projections to the mPFC supports behavioral studies that implicate adolescence as a sensitive window for risk-taking and cognitive flexibility in humans (van Duijvenvoorde et al., 2016; McCormick and Telzer, 2018) and in rodents (Johnson and Wilbrecht, 2011; Klune et al., 2021; Thies et al., 2025). Similarly, BLA^i^-to-mPFC afferents were nearly absent at neonatal age, emerged during the pre-juvenile period, and reached their highest density at juvenile age. Contralateral BLA and BMA afferents likewise peaked at juvenile age, indicating pronounced maturation of bilateral amygdala-prefrontal connectivity during this developmental window. This developmental trajectory has been previously described (Cunningham et al., 2002) and might be essential for the consolidation of fear memories. Notably, the BLA has also been shown to play a role in decision-making paradigms, in which effort and rewards values have to be evaluated (Floresco and Ghods-Sharifi, 2007). Moreover, the developmental increase in AM-derived (as part of the ATN^i^) mPFC-projecting neurons could facilitate dopamine-dependent rewards processing, as its role in reinforcing goal-directed behaviors has been shown in adult mice (Yang et al., 2022). Since the density of dopaminergic receptors in the mPFC peaks during adolescence (Larsen and Luna, 2018), the maturation of these inputs might contribute to the anatomical foundation for adaptive decision-making and behavioral flexibility.

While some brain areas maintained relatively stable densities of ipsilateral mPFC-projecting neurons after initial increase, other regions showed a decline of projections after early adolescence or even across the entire developmental period assessed here. RSP^i^ afferents peaked at pre-juvenile age before declining, SUM^i^ projections decreased after pre-juvenile age, and SUBd^i^ displayed an especially distinct trajectory, with dense neonatal projections that were nearly absent in adulthood. The RSP and SUM are involved in both prefrontal and hippocampal networks and play a role in spatial navigation as well as episodic memory (Vann et al., 2009; Ito et al., 2018; Loureiro et al., 2019).

Together with the transient developmental profiles of other cortical and limbic afferents, these findings raise the possibility that the mPFC receives a particularly broad and dense set of long-range inputs during the transition of young mice toward independent exploration. Such transient connectivity may facilitate developmental adaptations before being refined into the more selective organization characteristic of the adult prefrontal network.

### 4.3 Concluding remarks

Our data reveal that the development of mPFC afferents is neither linear nor uniform but characterized by region-specific developmental dynamics of afferent emergence, maintenance, and refinement (see Supplementary discussion). Moreover, quantitative comparison of ipsilateral and contralateral afferent labeling reveals a developmental shift from comparatively bilateral neonatal afferent labeling toward increasing ipsilateral dominance, which emerges through distinct mechanisms across individual afferent pathways. This refinement is accompanied by increasing myelination of interhemispheric prefrontal axons after neonatal age and by a particularly dense organization of several long-range inputs during the pre-juvenile and juvenile periods. These anatomical changes closely parallel known milestones of behavioral and functional maturation, suggesting structural development as a framework for functional circuit integration and adult-like cognitive functions.

At the same time, these developmental trajectories need to be interpreted within the methodological constraints of the present approach (see Supplementary discussion). Although injection coordinates and volumes were adjusted according to developmental brain size, injection spread was categorized, and viral spread was included as a covariate in the statistical models, these measures cannot fully exclude age-dependent differences in effective infection volume, viral uptake, retrograde transport efficiency, transgene expression, tissue properties, or the relative proportion of individual mPFC subdivisions targeted. Moreover, retrograde AAV efficiency has not been calibrated across the complete developmental range investigated here. Consequently, age-dependent differences in labeling should be interpreted as relative changes in the anatomical organization of mPFC afferents under matched experimental conditions rather than as absolute measures of connectivity or synaptic strength.

Within these interpretative boundaries, the present dataset nevertheless provides a quantitative framework for understanding how local, long-range, and interhemispheric afferents are reorganized during mPFC maturation and highlights developmental periods and pathways that may be particularly susceptible to perturbation. Knowledge about mPFC afferent development might help to understand how early afferent maturation shapes the integrative functions of the mPFC and how disruptions may result in lifelong phenotypes, such as those associated with neurodevelopmental disorders (Pöpplau and Hanganu-Opatz, 2024; Bitzenhofer and Günther, 2026).

## Data Availability

The original contributions presented in this study are included in this article/Supplementary material, further inquiries can be directed to the corresponding author.

## Footnotes

1
mouse.brain-map.org

## Supplementary material

Supplementary Table 1 Detailed statistical results.

Supplementary Table 2 Age-specific effects of sex and spread.

## References

[B1] Ährlund-Richter S. Xuan Y. van Lunteren J. A. Kim H. Ortiz C. Pollak Dorocic I. et al. (2019). A whole-brain atlas of monosynaptic input targeting four different cell types in the medial prefrontal cortex of the mouse. *Nat. Neurosci.* 22, 657–668. 10.1038/s41593-019-0354-y 30886408

[B2] Anastasiades P. G. Carter A. G. (2021). Circuit organization of the rodent medial prefrontal cortex. *Trends Neurosci.* 44, 550–563. 10.1016/j.tins.2021.03.006 33972100 PMC8222144

[B3] Bitzenhofer S. H. Günther A. (2026). Prefrontal spine loss in neuropsychiatric disorders: Circuit consequences for cognition. *Front. Psychiatry* 17:1730869. 10.3389/fpsyt.2026.1730869 42238096 PMC13226490

[B4] Brockmann M. D. Pöschel B. Cichon N. Hanganu-Opatz I. L. (2011). Coupled oscillations mediate directed interactions between prefrontal cortex and hippocampus of the neonatal rat. *Neuron* 71, 332–347. 10.1016/j.neuron.2011.05.041 21791291

[B5] Burgos-Robles A. Kimchi E. Y. Izadmehr E. M. Porzenheim M. J. Ramos-Guasp W. A. Nieh E. H. et al. (2017). Amygdala inputs to prefrontal cortex guide behavior amid conflicting cues of reward and punishment. *Nat. Neurosci.* 20, 824–835. 10.1038/nn.4553 28436980 PMC5448704

[B6] Chao O. Y. de Souza Silva M. A. Yang Y.-M. Huston J. P. (2020). The medial prefrontal cortex - hippocampus circuit that integrates information of object, place and time to construct episodic memory in rodents: Behavioral, anatomical and neurochemical properties. *Neurosci. Biobehav. Rev.* 113, 373–407. 10.1016/j.neubiorev.2020.04.007 32298711 PMC7302494

[B7] Chao O. Y. Huston J. P. Li J.-S. Wang A.-L. de Souza Silva M. A. (2016). The medial prefrontal cortex-lateral entorhinal cortex circuit is essential for episodic-like memory and associative object-recognition. *Hippocampus* 26, 633–645. 10.1002/hipo.22547 26501829

[B8] Chao O. Y. Nikolaus S. Lira Brandão M. Huston J. P. de Souza Silva M. A. (2017). Interaction between the medial prefrontal cortex and hippocampal CA1 area is essential for episodic-like memory in rats. *Neurobiol. Learn. Mem.* 141, 72–77. 10.1016/j.nlm.2017.03.019 28384498

[B9] Chini M. Hanganu-Opatz I. L. (2021). Prefrontal cortex development in health and disease: Lessons from rodents and humans. *Trends Neurosci.* 44, 227–240. 10.1016/j.tins.2020.10.017 33246578

[B10] Cunningham M. G. Bhattacharyya S. Benes F. M. (2002). Amygdalo-cortical sprouting continues into early adulthood: Implications for the development of normal and abnormal function during adolescence. *J. Comp. Neurol.* 453, 116–130. 10.1002/cne.10376 12373778

[B11] De Benedictis A. Petit L. Descoteaux M. Marras C. E. Barbareschi M. Corsini F. et al. (2016). New insights in the homotopic and heterotopic connectivity of the frontal portion of the human corpus callosum revealed by microdissection and diffusion tractography. *Hum. Brain Mapp.* 37, 4718–4735. 10.1002/hbm.23339 27500966 PMC6867471

[B12] de Faria O. Gonsalvez D. G. Nicholson M. Xiao J. (2019). Activity-dependent central nervous system myelination throughout life. *J. Neurochem.* 148, 447–461. 10.1111/jnc.14592 30225984 PMC6587454

[B13] De León Reyes N. S. Bragg-Gonzalo L. Nieto M. (2020). Development and plasticity of the corpus callosum. *Dev. Camb. Engl.* 147:dev189738. 10.1242/dev.189738 32988974

[B14] De León Reyes N. S. Mederos S. Varela I. Weiss L. A. Perea G. Galazo M. J. et al. (2019). Transient callosal projections of L4 neurons are eliminated for the acquisition of local connectivity. *Nat. Commun.* 10:4549. 10.1038/s41467-019-12495-w 31591398 PMC6779895

[B15] DeNardo L. A. Berns D. S. DeLoach K. Luo L. (2015). Connectivity of mouse somatosensory and prefrontal cortex examined with trans-synaptic tracing. *Nat. Neurosci.* 18, 1687–1697. 10.1038/nn.4131 26457553 PMC4624522

[B16] Ding S.-L. Yao Z. Hirokawa K. E. Nguyen T. N. Graybuck L. T. Fong O. et al. (2020). Distinct transcriptomic cell types and neural circuits of the subiculum and prosubiculum along the dorsal-ventral axis. *Cell Rep.* 31:107648. 10.1016/j.celrep.2020.107648 32433957 PMC7394561

[B17] Fitsiori A. Nguyen D. Karentzos A. Delavelle J. Vargas M. I. (2011). The corpus callosum: White matter or terra incognita. *Br. J. Radiol.* 84, 5–18. 10.1259/bjr/21946513 21172964 PMC3473808

[B18] Floresco S. B. Ghods-Sharifi S. (2007). Amygdala-Prefrontal cortical circuitry regulates effort-based decision making. *Cereb. Cortex* 17, 251–260. 10.1093/cercor/bhj143 16495432

[B19] Fornari E. Knyazeva M. G. Meuli R. Maeder P. (2007). Myelination shapes functional activity in the developing brain. *NeuroImage* 38, 511–518. 10.1016/j.neuroimage.2007.07.010 17889561

[B20] Fyhn M. Molden S. Hollup S. Moser M.-B. Moser E. (2002). Hippocampal neurons responding to first-time dislocation of a target object. *Neuron* 35, 555–566. 10.1016/s0896-6273(02)00784-5 12165476

[B21] Günther A. Chini M. Bitzenhofer S. H. Marquardt A. Hanganu-Opatz I. L. (2026). Early rebalancing of neuroinflammatory cascades lastingly rescues prefrontal deficits in a 22q11.2ds model. *Brain Behav. Immun.* 134:106470. 10.1016/j.bbi.2026.106470 41621631

[B22] Hanganu-Opatz I. L. Klausberger T. Sigurdsson T. Nieder A. Jacob S. N. Bartos M. et al. (2023). Resolving the prefrontal mechanisms of adaptive cognitive behaviors: A cross-species perspective. *Neuron* 111, 1020–1036. 10.1016/j.neuron.2023.03.017 37023708

[B23] Heidbreder C. A. Groenewegen H. J. (2003). The medial prefrontal cortex in the rat: Evidence for a dorso-ventral distinction based upon functional and anatomical characteristics. *Neurosci. Biobehav. Rev.* 27, 555–579. 10.1016/j.neubiorev.2003.09.003 14599436

[B24] Honjoh S. Sasai S. Schiereck S. S. Nagai H. Tononi G. Cirelli C. (2018). Regulation of cortical activity and arousal by the matrix cells of the ventromedial thalamic nucleus. *Nat. Commun.* 9:2100. 10.1038/s41467-018-04497-x 29844415 PMC5974306

[B25] Hoover W. B. Vertes R. P. (2007). Anatomical analysis of afferent projections to the medial prefrontal cortex in the rat. *Brain Struct. Funct.* 212, 149–179. 10.1007/s00429-007-0150-4 17717690

[B26] Innocenti G. M. Schmidt K. Milleret C. Fabri M. Knyazeva M. G. Battaglia-Mayer A. et al. (2022). The functional characterization of callosal connections. *Prog. Neurobiol.* 208:102186. 10.1016/j.pneurobio.2021.102186 34780864 PMC8752969

[B27] Ito H. T. Moser E. I. Moser M.-B. (2018). Supramammillary nucleus modulates spike-time coordination in the prefrontal-thalamo-hippocampal circuit during navigation. *Neuron* 99, 576–587.e5. 10.1016/j.neuron.2018.07.021 30092214

[B28] Johnson C. Wilbrecht L. (2011). Juvenile mice show greater flexibility in multiple choice reversal learning than adults. *Dev. Cogn. Neurosci.* 1, 540–551. 10.1016/j.dcn.2011.05.008 21949556 PMC3177141

[B29] Klune C. B. Jin B. DeNardo L. A. (2021). Linking mPFC circuit maturation to the developmental regulation of emotional memory and cognitive flexibility. *eLife* 10:e64567. 10.7554/eLife.64567 33949949 PMC8099425

[B30] Kostka J. K. Bitzenhofer S. H. (2022). Postnatal Development of Centrifugal Inputs to the Olfactory Bulb. *Front. Neurosci.* 16:815282. 10.3389/fnins.2022.815282 35281496 PMC8908425

[B31] Larsen B. Luna B. (2018). Adolescence as a neurobiological critical period for the development of higher-order cognition. *Neurosci. Biobehav. Rev.* 94, 179–195. 10.1016/j.neubiorev.2018.09.005 30201220 PMC6526538

[B32] Laubach M. Amarante L. M. Swanson K. White S. R. (2018). What, If Anything, Is Rodent Prefrontal Cortex? *eNeuro* 5:ENEURO.0315-18.2018. 10.1523/ENEURO.0315-18.2018 30406193 PMC6220587

[B33] Loureiro M. Achargui R. Flakowski J. Van Zessen R. Stefanelli T. Pascoli V. et al. (2019). Social transmission of food safety depends on synaptic plasticity in the prefrontal cortex. *Science* 364, 991–995. 10.1126/science.aaw5842 31171697

[B34] MacDonald C. J. Lepage K. Q. Eden U. T. Eichenbaum H. (2011). Hippocampal “Time Cells” bridge the gap in memory for discontiguous events. *Neuron* 71, 737–749. 10.1016/j.neuron.2011.07.012 21867888 PMC3163062

[B35] Maren S. Quirk G. J. (2004). Neuronal signalling of fear memory. *Nat. Rev. Neurosci.* 5, 844–852. 10.1038/nrn1535 15496862

[B36] McCormick E. M. Telzer E. H. (2018). Not doomed to repeat: Enhanced medial prefrontal cortex tracking of errors promotes adaptive behavior during adolescence. *J. Cogn. Neurosci.* 30, 281–289. 10.1162/jocn_a_01206 29131744 PMC5797691

[B37] Milardi D. Bramanti P. Milazzo C. Finocchio G. Arrigo A. Santoro G. et al. (2015). Cortical and subcortical connections of the human claustrum revealed in vivo by constrained spherical deconvolution tractography. *Cereb. Cortex* 25, 406–414. 10.1093/cercor/bht231 24014669

[B38] Minciacchi D. Granato A. (1989). Development of the thalamocortical system: Transient-crossed projections to the frontal cortex in neonatal rats. *J. Comp. Neurol.* 281, 1–12. 10.1002/cne.902810102 2925895

[B39] Numan R. (2015). A prefrontal-hippocampal comparator for goal-directed behavior: The intentional self and episodic memory. *Front. Behav. Neurosci.* 9:323. 10.3389/fnbeh.2015.00323 26635567 PMC4658443

[B40] Pachitariu M. Stringer C. (2022). Cellpose 2.0: How to train your own model. *Nat. Methods* 19, 1634–1641. 10.1038/s41592-022-01663-4 36344832 PMC9718665

[B41] Parbst F. Kostka J. K. Günther A. Chen Y.-N. Hanganu-Opatz I. L. Bitzenhofer S. H. (2025). Developmental olfactory dysfunction and abnormal odor memory in immune-challenged Disc1+/- Mice. *J. Neurosci.* 45:e1007242025. 10.1523/JNEUROSCI.1007-24.2025 40447445 PMC12178285

[B42] Park Y.-G. Sohn C. H. Chen R. McCue M. Yun D. H. Drummond G. T. et al. (2019). Protection of tissue physicochemical properties using polyfunctional crosslinkers. *Nat. Biotechnol.* 37, 73–83. 10.1038/nbt.4281 30556815 PMC6579717

[B43] Parnaudeau S. Bolkan S. S. Kellendonk C. (2018). The mediodorsal thalamus: An essential partner of the prefrontal cortex for cognition. *Biol. Psychiatry* 83, 648–656. 10.1016/j.biopsych.2017.11.008 29275841 PMC5862748

[B44] Paus T. Zijdenbos A. Worsley K. Collins D. L. Blumenthal J. Giedd J. N. et al. (1999). Structural maturation of neural pathways in children and adolescents: In vivo study. *Science* 283, 1908–1911. 10.1126/science.283.5409.1908 10082463

[B45] Paxinos G. (2007). *Atlas of the Developing Mouse Brain at E17.5, P0, and P6.* Academic Press.

[B46] Pöpplau J. A. Hanganu-Opatz I. L. (2024). Development of prefrontal circuits and cognitive abilities. *Cold Spring Harb. Perspect. Biol.* 16:a041502. 10.1101/cshperspect.a041502 38692836 PMC11444252

[B47] Pöpplau J. A. Schwarze T. Dorofeikova M. Pochinok I. Günther A. Marquardt A. et al. (2024). Reorganization of adolescent prefrontal cortex circuitry is required for mouse cognitive maturation. *Neuron* 112, 421–440.e7. 10.1016/j.neuron.2023.10.024 37979584 PMC10855252

[B48] Rios O. Villalobos J. (2004). Postnatal development of the afferent projections from the dorsomedial thalamic nucleus to the frontal cortex in mice. *Brain Res. Dev. Brain Res.* 150, 47–50. 10.1016/j.devbrainres.2004.02.005 15126037

[B49] Song L. Xu X. Putthoff P. Fleck D. Spehr M. Hanganu-Opatz I. L. (2022). Sparser and less efficient hippocampal-prefrontal projections account for developmental network dysfunction in a model of psychiatric risk mediated by gene-environment interaction. *J. Neurosci.* 42, 601–618. 10.1523/JNEUROSCI.1203-21.2021 34844990 PMC8805616

[B50] Spellman T. Rigotti M. Ahmari S. E. Fusi S. Gogos J. A. Gordon J. A. (2015). Hippocampal–prefrontal input supports spatial encoding in working memory. *Nature* 522, 309–314. 10.1038/nature14445 26053122 PMC4505751

[B51] Sturrock R. R. (1980). Myelination of the mouse corpus callosum. *Neuropathol. Appl. Neurobiol.* 6, 415–420. 10.1111/j.1365-2990.1980.tb00219.x 7453945

[B52] Szczupak D. Iack P. M. Liu C. IRC5 Consortium, Tovar-Moll F. Lent R. et al. (2021). Direct interhemispheric cortical communication via thalamic commissures: A new white-matter pathway in the rodent brain. *Cereb. Cortex* 31, 4642–4651. 10.1093/cercor/bhab112 33999140 PMC8408456

[B53] Szczupak D. Iack P. M. Rayêe D. Liu C. Lent R. Tovar-Moll F. et al. (2023). The relevance of heterotopic callosal fibers to interhemispheric connectivity of the mammalian brain. *Cereb. Cortex* 33, 4752–4760. 10.1093/cercor/bhac377 36178137 PMC10110439

[B54] Takehara-Nishiuchi K. (2014). Entorhinal cortex and consolidated memory. *Neurosci. Res.* 84, 27–33. 10.1016/j.neures.2014.02.012 24642278

[B55] Thies A. M. Pochinok I. Marquardt A. Dorofeikova M. Hanganu-Opatz I. L. Pöpplau J. A. (2025). Trajectories of working memory and decision making abilities along juvenile development in mice. *Front. Neurosci.* 19:1524931. 10.3389/fnins.2025.1524931 40092072 PMC11906447

[B56] van der Knaap L. J. van der Ham I. J. M. (2011). How does the corpus callosum mediate interhemispheric transfer? *A review. Behav. Brain Res.* 223, 211–221. 10.1016/j.bbr.2011.04.018 21530590

[B57] van Duijvenvoorde A. C. K. Peters S. Braams B. R. Crone E. A. (2016). What motivates adolescents? Neural responses to rewards and their influence on adolescents’ risk taking, learning, and cognitive control. *Neurosci. Biobehav. Rev.* 70, 135–147. 10.1016/j.neubiorev.2016.06.037 27353570

[B58] Vann S. D. Aggleton J. P. Maguire E. A. (2009). What does the retrosplenial cortex do? *Nat. Rev. Neurosci.* 10, 792–802. 10.1038/nrn2733 19812579

[B59] Voigt F. F. Kirschenbaum D. Platonova E. Pagès S. Campbell R. A. A. Kastli R. et al. (2019). The mesoSPIM initiative: Open-source light-sheet microscopes for imaging cleared tissue. *Nat. Methods* 16, 1105–1108. 10.1038/s41592-019-0554-0 31527839 PMC6824906

[B60] Wang Y. Chen Z. Ma G. Wang L. Liu Y. Qin M. et al. (2023). A frontal transcallosal inhibition loop mediates interhemispheric balance in visuospatial processing. *Nat. Commun.* 14:5213. 10.1038/s41467-023-40985-5 37626171 PMC10457336

[B61] Yang C. Hu Y. Talishinsky A. D. Potter C. T. Calva C. B. Ramsey L. A. et al. (2022). Medial prefrontal cortex and anteromedial thalamus interaction regulates goal-directed behavior and dopaminergic neuron activity. *Nat. Commun.* 13:1386. 10.1038/s41467-022-28892-7 35296648 PMC8927595

[B62] Yorke C. H. Jr. Caviness V. S. Jr. (1975). Interhemispheric neocortical connections of the corpus callosum in the normal mouse: A study based on anterograde and retrograde methods. *J. Comp. Neurol.* 164, 233–245. 10.1002/cne.901640206 1184784

