# Supplementary material for: Mapping prefrontal afferents along development
Source: Front Neurosci. 2026 Sep 16;20:1861524. doi: 10.3389/fnins.2026.1861524 (PMC13623852; doi:10.3389/fnins.2026.1861524)
Supplement: Supplementary file 1 [file Supplementary_file_1.pdf]

Mapping prefrontal afferents along development

Anne Günther[1*], Malte Stelzer[1*], Andreas Franzelin[2], Annette Marquardt[1], Thomas Oertner[2], Ileana Hanganu-Opatz[1#], Jastyn A. Pöpplau[1#]

Inventory of Supplementary Information

**Inventory of Supplementary Information**	Page
Supplementary discussion	3
Figure S1	5
Figure S2	6

SUPPLEMENTARY DISCUSSION

Local mPFC connectivity

Local prefrontal circuits rely on input from long-range projections in order to process incoming information and execute prefrontal functions (Hanganu-Opatz et al., 2023). We observed slight regional specificity of mPFC projection dynamics. While prefrontal afferent labeling from PL^{i} and ILA^{i} increased from neonatal to adult age, the ACA^{i} showed an increase towards pre-juvenile age but no difference between neonatal and adult age. Notably, the PL^{c} was the only prefrontal subdivision that showed an increase in prefrontal afferent labeling from neonatal to adult age. In contrast, the ORB^{c} showed a decline in contralateral projections. These divergent trajectories potentially reflect distinct cytoarchitectural differentiation between prefrontal areas during maturation of callosal connectivity (Barbas et al., 2005; Kolk and Rakic, 2022), possibly reflecting their contributions to cognitive, emotional, and sensorimotor processing. On a functional level, previous work identified a peak in broadband gamma and spiking activity in the mPFC during early adolescence which orchestrates a functional reorganization of mPFC circuits. This period of heightened activity is followed by a reduction, ultimately leading to mature mPFC synchrony at adult age (Pöpplau et al., 2024). Thus, the temporal rise in external limbic afferents may serve as instructive signals for the refinement of mPFC microcircuits.

Methodological Considerations

The retrograde labeling virus rAAV9rg_CaMKII-mCherry does not exclusively infect excitatory neurons via axonal pathways but can also infect neurons in close proximity via their somata. Therefore, quantification of labeled cells, especially in subdivisions of the ipsilateral mPFC could have been biased by an uneven distribution of the retrograde labeling virus. Furthermore, the only identified increase in interhemispheric afferent labeling was observed in the PL^{c}, which could be explained by a bias due to experimental procedures. Homotopic regions are more strongly connected to each other than heterotopic ones (Barbas et al., 2005) and as the PL is located between the ACA and ILA on the dorso-ventral axis, it is most likely that the PL^{i} received the largest portion of viral vector during the injection procedures, resulting in the PL^{c} being labeled more strongly than the heterotopic ACA^{c} and ILA^{c}.

Notably, labeling in several contralateral regions was influenced by viral spread, whereas among ipsilateral afferents such an effect was detected only for the ORB. Given the generally lower abundance and spatially heterogeneous organization of many contralateral afferent populations, their quantitative assessment may be particularly sensitive to differences in the extent and precise location of the injection site (Ährlund-Richter et al., 2019, DeNardo et al. 2015). Thus, variation in viral spread may recruit proportionally different populations of contralaterally projecting neurons. Explicitly accounting for viral spread in the statistical models allowed age-dependent developmental effects to be assessed independently of this variability.

An additional limitation arises from comparing retrograde viral labeling across different developmental stages. Despite age-adjusted injection volumes and coordinates, developmental changes in tissue composition, axonal maturation, viral uptake, retrograde transport, or transgene expression may influence labeling efficiency independently of the underlying anatomical projection. Thus, the present data are best interpreted as relative developmental trajectories obtained under standardized experimental conditions rather than as absolute measures of projection number or strength.

Besides viral spread, our statistical analyses revealed region-specific effects of sex on several afferent populations. Females showed lower projection densities from CLAi, RSPi, PIRIi, and SUMi across age groups, whereas VMi projections showed the opposite pattern after neonatal age, with higher densities in females. The absence of a uniform direction across regions argues against a general sex-dependent difference in retrograde labeling and instead suggests that sex selectively influences the maturation or organization of specific prefrontal afferent pathways. The functional significance of these anatomical differences remains to be determined.

Finally, because all injections were performed in the right mPFC, potential hemispheric asymmetries in individual afferent pathways cannot be excluded; however, the use of a consistent injection hemisphere across all age groups minimizes this factor as a source of variability in the developmental comparisons.

Notes on Specific Brain Areas

We included the quantification of very sparsely labeled cells from the midbrain in our analysis, which have not been reported to project to the adult mPFC via excitatory glutamatergic fibers in previous studies. Prior research shows that the PAG receives projections from the mPFC (Anastasiades and Carter, 2021), but no study provides evidence for reciprocal connectivity. The VTA is known to innervate the mPFC via dopaminergic connections (Tzschentke, 2000), but glutamatergic projections from the VTA to the mPFC have not been described before. The absence of reports of glutamatergic prefrontal afferents from the PAG and VTA may result from the very low number of prefrontally projecting cells and/or the transient nature of these projections during development, as our results suggest. Finally, in our analysis we could not detect any projections from the SUBd to the mPFC after neonatal age, while a previous study described prefrontal afferents from the SUBd in adult animals (Strange et al., 2014) but without identifying such projections consistently for all experimental subjects. The cause of such conflicting results remains unclear and requires further experimental research.

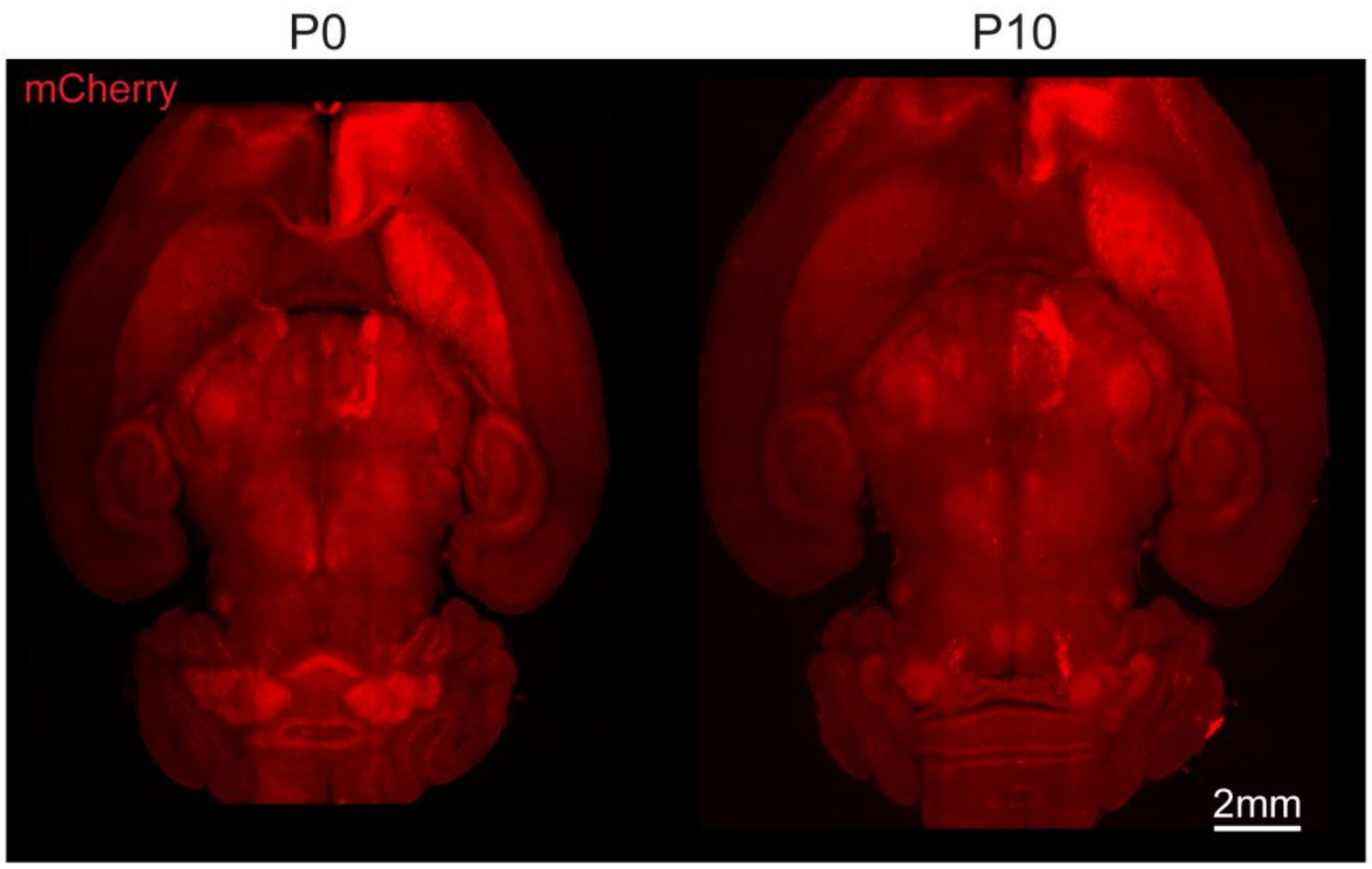

FIGURE S1 related to FIGURE 1 | Light-sheet microscopy during early development. Representative light-sheet microscopy images of a transverse section of a neonatal (left, injected at P0, perfused at P10) and pre-juvenile (right, injected at P10, perfused at P20) mouse with retrograde mCherry labeling (red).

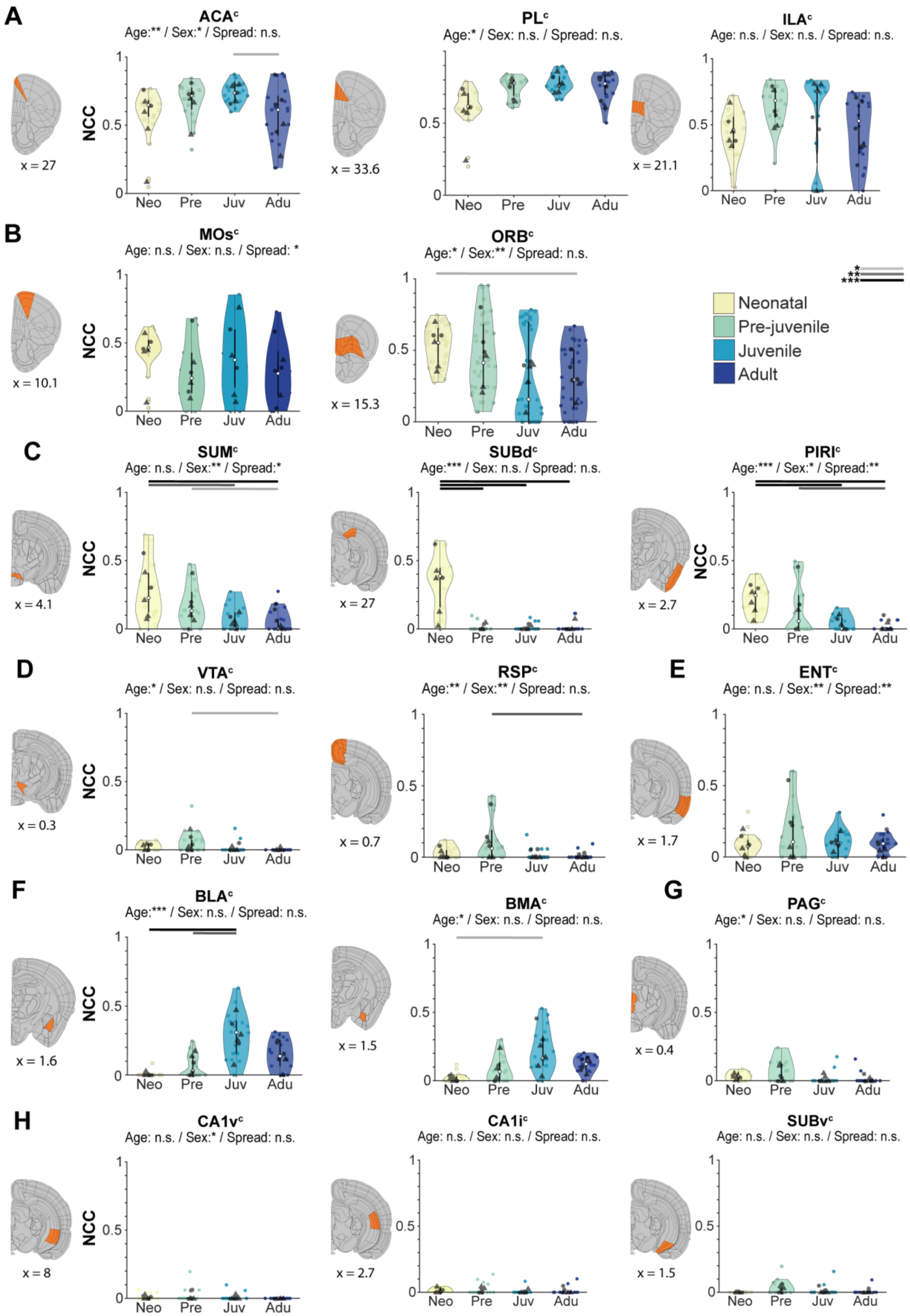
A
ACAᶜ
Age:** / Sex:* / Spread: n.s.
x = 27
PLᶜ
Age:* / Sex: n.s. / Spread: n.s.
x = 33.6
ILAᶜ
Age: n.s. / Sex: n.s. / Spread: n.s.
x = 21.1
B
MOsᶜ
Age: n.s. / Sex: n.s. / Spread: *
x = 10.1
ORBᶜ
Age:* / Sex:** / Spread: n.s.
x = 15.3
*
**
***
Neonatal
Pre-juvenile
Juvenile
Adult
C
SUMᶜ
Age: n.s. / Sex:** / Spread:*
x = 4.1
SUBdᶜ
Age:*** / Sex: n.s. / Spread: n.s.
x = 27
PIRIᶜ
Age:*** / Sex:* / Spread:**
x = 2.7
D
VTAᶜ
Age:* / Sex: n.s. / Spread: n.s.
x = 0.3
RSPᶜ
Age:** / Sex:** / Spread: n.s.
x = 0.7
E
ENTᶜ
Age: n.s. / Sex:** / Spread:**
x = 1.7
F
BLAᶜ
Age:*** / Sex: n.s. / Spread: n.s.
x = 1.6
BMAᶜ
Age:* / Sex: n.s. / Spread: n.s.
x = 1.5
G
PAGᶜ
Age:* / Sex: n.s. / Spread: n.s.
x = 0.4
H
CA1vᶜ
Age: n.s. / Sex:* / Spread: n.s.
x = 8
CA1iᶜ
Age: n.s. / Sex: n.s. / Spread: n.s.
x = 2.7
SUBvᶜ
Age: n.s. / Sex: n.s. / Spread: n.s.
x = 1.5
NCC
0
0.5
1
Neo
Pre
Juv
Adu

FIGURE S2 related to FIGURE 5 | Age-dependent dynamics of contralateral projections to the mPFC. **(A)** Left, Violin plots displaying the NCC of prefrontally projecting ACA^{c} cells (n = 72 images, 23 mice). Middle, same as left for PL^{c} (n = 72 images, 23 mice) . Right, same as left for ILA^{c} (n = 80 images, 24 mice). **(B)** Left, same as (A) for MOs^{c} (n = 70 images, 24 mice). Right, for ORB^{c} (n = 139 images, 24 mice). **(C)** Left, same as (A) for SUM^{c} (n = 65 images, 24 mice). Middle, same as (A) for SUBd^{c} (n = 68 images, 24 mice). Right, same as (A) for PIRI^{c} (n = 63 images, 22 mice). **(D)** Left, same as (A) for VTA^{c} (n = 72 images, 23 mice). Right, same as (A) for RSP^{c} (n = 71 images, 24 mice). **(E)** Same as (A) for ENTc (n = 63 images, 23 mice). **(F)** Left, same as (A) for BLA^{c} (n = 70 images, 24 mice). Right, same as (A) for BMA^{c} (n = 71 images, 24 mice). **(G)** Same as (A) for PAG^{c} (n = 67 images, 24 mice). (H) Left, same as (A) for CA1v^{c} (n = 132 images, 24 mice). Middle, same as (A) for CA1i^{c} (n = 65 images, 24 mice). Right, same as (A) for SUBv^{c} (n = 65 images, 24 mice). Small colored dots indicate individual image-level measurements, whereas larger black circles and triangles indicate the mean NCC of individual male and female mice, respectively. Violin plots are represented as median with 25th to 75th percentile. See Statistics table S1 for details. Significant differences are indicated as *, **, *** for $p<0.05$, 0.01, 0.001, respectively.
